# History of Graduate Education in Nursing and the Development of Technological Products in the Professional Master’s Program

**DOI:** 10.1590/0034-7167-2025-0203

**Published:** 2026-08-21

**Authors:** Pedro Leite de Melo, Alessandra Matheus Domingos, Daiana Kloh Khalaf, Marina de Góes Salvetti, Maria do Perpétuo Socorro de Sousa Nóbrega

**Affiliations:** I Universidade de São Paulo. São Paulo, São Paulo, Brazil; II Universidade Federal do Paraná. Curitiba, Paraná, Brazil

**Keywords:** Postgraduate Education in Nursing, Development of Technological Products, Scientific Research, Nursing, Innovation., Educación de Postgrado en Enfermería, Desarrollo de Productos Tecnológicos, Investigación Científica, Enfermería, Innovación.

## Abstract

**Objectives:**

to reflect on the trajectory and on the technical and technological production of the Professional Master’s Degree in Nursing.

**Methods:**

a theoretical reflective study developed from a bibliographic and documentary survey conducted between September and November 2024, through searches in the Medical Literature Analysis and Retrieval System Online, Latin American and Caribbean Health Sciences Literature, Scientific Electronic Library Online, Web of Science, Excerpta Medica Database, and through the collection of official documents from the Brazilian Ministry of Education related to the evaluation of Graduate Programs, as well as information from the CAPES website and the Sucupira Platform.

**Results:**

after reflective reading and organization of the selected material, the category Historical trajectory, technical and technological production of the Professional Master’s Degree in Nursing emerged.

**Final Considerations:**

Professional Master’s Degrees in Nursing still face challenges in strengthening their identity/objective, the methodological process of product development, implementation, and impact on health services.

## RESUMEN

**Objetivos:**

reflexionar sobre la trayectoria y la producción técnica y tecnológica del Máster Profesional en Enfermería.

**Métodos:**

estudio teórico reflexivo desarrollado a partir de un levantamiento bibliográfico y documental identificado entre septiembre y noviembre de 2024, mediante búsquedas en las bases *Medical Literature Analysis and Retrieval System Online*, Literatura Latinoamericana y del Caribe en Ciencias de la Salud, *Scientific Electronic Library Online, Web of Science, Excerpta Medica Database*, y documentalmente a partir de la recopilación de documentos oficiales del Ministerio de Educación de Brasil, relacionados con las evaluaciones de los Programas de Posgrado, así como de informaciones del sitio web de CAPES y de la Plataforma Sucupira.

**Resultados:**

tras la lectura reflexiva y la organización del material seleccionado, emergió la categoría Trayectoria histórica, producción técnica y tecnológica del Máster Profesional en Enfermería.

**Consideraciones Ffinales:**

los Másteres Profesionales en Enfermería aún enfrentan desafíos para su fortalecimiento en cuanto a su identidad/objetivo, el proceso metodológico de construcción de productos, su implementación y el impacto en los servicios de salud.

## INTRODUCTION

The Professional Master’s Degree in Nursing (PMN) was introduced in Brazil in the late 1990s with the aim of training highly qualified nurses capable of conducting advanced and transformative practice to meet social, organizational, and contemporary labor market demands^(1)^.

Graduate education programs (*stricto sensu*), since its inception, has exerted a fundamental influence across all fields of knowledge, including nursing, contributing significantly to the evolution of the profession as a science and, consequently, to the improvement of nurses’ clinical practice. This advancement and transformation in professional practice are intrinsically linked to educational processes and professional qualification. In this sense, master’s and doctoral training not only expands the theoretical foundation of nurses but also develops essential practical and strategic competencies needed to address current challenges in the field of nursing^(2)^.

The National Graduate Plan (2011-2020) recorded a significant expansion of Professional Master’s Programs in Nursing, reaching 24 programs in 2019 and approving two professional doctoral programs. However, despite this progress, investment in technological innovation remains limited, affected by public sector budget cuts that impact the funding of graduate programs^(3)^.

Another point to be discussed is the fact that nurses often lack adequate technical and scientific knowledge to conduct research on technological productivity, due to insufficient training in this area and the difficulty of developing studies that result in technological production, since this represents a relatively new model of intellectual output in nursing^(4)^.

In both personal and professional daily life, nurses are constantly in contact with new technologies, and the faster they assimilate and master these innovations to apply them for the benefit of human beings, the greater the advances in their professional practice will be. In this context, it is essential that nurses have access to and make use of scientific and technological research in order to contribute to improving the quality of care provided^(5)^.

The PMN is not limited to training the graduate student; it also requires the development of scientific investigation related to their field of practice. However, it is not enough to merely analyze the challenges of professional practice - it is essential that these studies result in solution proposals, materialized in different forms of technological production^(6)^.

Technological or technical production refers to the work developed by permanent faculty members and students that does not fall under the category of scientific production. Its value is recognized through the interaction between academia and the community, resulting in practical and innovative applications that address real demands and needs^(7)^.

## OBJECTIVES

To reflect on the trajectory and on the technical and technological production of the Professional Master’s Degree in Nursing.

## METHODS

A theoretical reflective study on the historical trajectory of PMN, beginning with Ordinance No. 80/16/12/1998, issued by the *Coordenação de Aperfeiçoamento de Pessoal de Nível Superior* (*CAPES*, Coordination for the Improvement of Higher Education Personnel), which establishes the guidelines for their creation and operation^(8)^.

The bibliographic material was identified between September and November 2024 through searches in the MEDLINE/PubMed (Medical Literature Analysis and Retrieval System Online), LILACS (Latin American and Caribbean Health Sciences Literature), SciELO (Scientific Electronic Library Online), WoS (Web of Science), and EMBASE (Excerpta Medica Database) databases, using the following descriptors: Graduate Education in Nursing, Development of Technological Products, Scientific Research, Nursing, and Innovation. The documentary material was identified from official documents of the Ministry of Education related to the evaluation of Graduate Programs, as well as information available on the CAPES website (https://www.gov.br/capes/pt-br) and the Sucupira Platform (https://sucupira.capes.gov.br/), during the same period.

The information extracted from the studies was organized by the authors of this reflection throughout the entire process of selecting, reading, and analyzing the texts, followed by the development of theoretical reflective notes on the main points for decision making and analytical interpretation.

The authors’ experiences with the topic, supported by the theoretical framework on the challenges encountered in consolidating and characterizing the technical and technological products of PMN, together with a careful reading aimed at identifying information about their trajectory, enabled the development of the study.

The reflections presented were developed based on the theoretical and conceptual aspects addressed in the studies, as well as on the authors’ impressions derived from their understanding of the topic and empirical experience. As the study did not involve data collection with human participants, ethical review was not required. However, procedures were adopted to ensure that the primary studies used were ethically respected.

## RESULTS

### Historical trajectory, technical and technological production of the Professional Master’s Degree in Nursing

*CAPES* classifies Professional Master’s Programs in Nursing as a *stricto sensu* graduate modality aimed at professional training in various fields of knowledge through the study of techniques, processes, and themes aligned with labor market demands^(9)^. Based on Ordinance No. 80 of December 16, 1998, the first Professional Master’s Program in Nursing in the country was created in 2001, with a focus on obstetrics and linked to the Federal University of São Paulo. After graduating four master’s students, the program ended its activities in 2004.

Chart 1 illustrates the development of Professional Master’s Programs in Nursing from 2003 to 2019, mapping 24 programs according to the CAPES 2017-2020 Report^(10)^.

**Chart 1:** Timeline of Professional Master’s Degrees in Nursing from 2003 to 2019, São Paulo, São Paulo, Brazil

Graduate Program Year of Creation	Higher Education Institution	Program Concentration Area
Professional Master’s Program in Assistential Nursing - 2003	*Universidade Federal Fluminense*	Nursing Care Processes
Graduate Program in Nursing - 2006	*Universidade Estadual Paulista Júlio de Mesquita Filho em Botucatu*	Nursing
Nursing Care Management - 2010	*Universidade Federal de Santa Catarina*	Management of Health and Nursing Care
Graduate Program in Nursing - 2011	*Universidade Federal do Paraná*	Professional Nursing Practice
Graduate Program in Nursing - 2011	*Universidade Federal do Espírito Santo*	Care and Health Administration
Graduate Program in Nursing - 2011	*Universidade do Vale do Rio dos Sinos*	Nursing Care Practices
Graduate Program in Family Health - 2011	*Centro Universitário UNINOVAFAPI*	Family Health
Graduate Program in Nursing - 2012	*Universidade Estadual de Feira de Santana*	Nursing
Graduate Program in Health Sciences - 2012	*Fund. Ensino e Pesquisa em Ciências de Saúde*	Quality of Health Care
Graduate Program in Health and Technology in the Hospital Environment - 2013	*Universidade Federal do Estado do Rio de Janeiro*	Health and Technology in the Hospital Setting
Graduate Program in Nursing Technology and Innovation - 2013	*Universidade Federal de São Paulo/Ribeirão Preto*	Caring Technologies in Nursing
Graduate Program in Nursing in Primary Health Care within the SUS - 2013	*Universidade de São Paulo*	Care in Primary Health Care
Graduate Program in Nursing - 2014	*Sociedade Beneficente Israelita Brasileira Albert Einstein*	Teaching and Health
Graduate Program in Family Health - 2015	*Faculdade Nova Esperança*	Management and Technologies of Health Care
Graduate Program in Nursing - 2015	*Universidade Federal de Ciências da Saúde de Porto Alegre*	Nursing
Graduate Program in Maternal and Child Health - 2015	*Universidade Franciscana*	Maternal and Child Health
Graduate Program in Nursing Technology and Innovation - 2016	*Universidade de Fortaleza*	Caring Technologies in Nursing
Graduate Program in Gerontology - 2016	*Universidade Federal da Paraíba*	Gerontology
Graduate Program in Health Informatics - 2016	*Universidade Federal de Santa Catarina*	Health Informatics
Graduate Program in Nursing in Primary Health Care - 2017	*Universidade do Estado de Santa Catarina*	Health Promotion in Primary Care
Graduate Program in Health and Society - 2017	*Universidade Federal Rio Grande do Norte*	Knowledge and Practices in Health and Education
Graduate Program in Public Health Nursing - 2019	*Universidade do Estado do Amazonas*	Nursing Practices in Public Health in the Amazon
Graduate Program in Nursing in the Amazon Context - 2019	*Universidade Federal do Amazonas*	Advanced Clinical Practice in Amazonian Nursing
Graduate Program in Nursing - 2019	*Universidade do Estado de Santa Catarina*	Nursing

Since 2014, the *Conselho Federal de Enfermagem* (*COFEN*, Federal Nursing Council) has included in its triennial plans funding proposals for Professional Master’s Programs aimed at nurses working in the *Sistema Único de Saúde* (*SUS*, Brazilian Unified Health System), with the goal of improving the quality of health care^(9)^. In mid 2016, it launched the first public call for the PMN and allocated resources to public and private educational institutions interested in promoting research in the area of Nursing Care Systematization.

In the following year, *CAPES* and *COFEN* established a joint evaluation committee that selected 16 programs and distributed 140 positions. Additionally, during the 20th, 21st, and 22nd Brazilian Congresses of Nursing Councils, held between 2017 and 2019, roundtable discussions on the PMN were included in the scientific program to address aspects of its functioning^(9)^.

The technical/technological output of the PMN was evaluated for the first time in the four year assessment cycle of the programs (2013-2016) regarding its typology, and the results showed that challenges remain in classifying this production in a way that ensures it effectively contributes to the quality of health services^(10)^.

The PMN is in a process of consolidation, particularly regarding the development and improvement of technical and technological production. There are still difficulties in classifying its products, and in most cases it is necessary to evaluate the material in full in order to understand its typology and categorize it correctly. Another aspect related to the qualification of the PMN concerns its identity, objectives, methodological process for constructing products and implementing them, as well as the impact they generate on health services^(11)^.

Before 2012, there was no technological *Qualis* system to evaluate the PMN’s output. The assessment was based on the criteria and metrics used for Academic Master’s programs, which focus on intellectual production^(6)^. To address this issue, CAPES developed in 2012 a guidance framework for the Advisory Committee to improve the information provided in the Sucupira Platform, covering six components: program proposal, faculty, student body, theses and dissertations/final papers, intellectual production, and social impact^(7)^.

With the growth of the PMN over recent years, a significant number of technical and technological products have been developed by nurses, with the aim of offering innovations for practice, ensuring safety and quality in care, and valuing professional expertise^(5)^.

To address this challenge and strengthen the PMN, the Evaluation Directorate of CAPES established in 2016 the Technical and Technological Qualis Working Group (“*GT PTT*”). This group gathered 64 technical productions available on the Sucupira Platform (2013-2016 quadrennium), analyzed them, and defined 21 types of Technical and Technological Production, which were approved in 2019 by the Higher Education Technical Scientific Council^(10)^, as shown in Chart 2.

**Chart 2:** Characterization of Technical/Technological Productions, São Paulo, São Paulo, Brazil

Type of technical production and Definition	Exemples
Bibliographic Product: Aimed at disseminating knowledge produced within academic institutions.	Articles published in technical journals; reviews or critiques; articles in newspapers or outreach magazines.
Intellectual Property Assets: Resources that add value to brands and developed products, while ensuring copyright protection.	Invention patents, utility model patents, certificates of addition; trademarks, industrial designs, geographical indications; integrated circuit topographies.
Social Technology: Methods, processes, or products developed and applied with the population to improve living conditions in the community. Must be simple, low-cost, and easy to apply.	Reading projects at bus terminals; alternative agricultural techniques; oral health education for specific population groups.
Professional Training Course: A set of content organized according to the competencies required for professional training, aligned with the objectives of the Graduate Program.	Continuing education for professionals with institutional ties; special offerings for professionals involved in research projects.
Publishing Product: Result of editorial activities involving the editing and publication of fiction and non-fiction works.	Printed media (newspapers, magazines, books, pamphlets, posters), electronic media (e-books, interactive media), or digital media (internet, mobile).
Didactic Material: Support materials used for teaching and learning processes in different educational contexts.	Printed collections; textbooks and supplementary books; guides; thematic maps; educational games.
Software/Application (Computer Program): A set of instructions or statements used directly or indirectly by a computer to obtain a specific result.	Simulation programs; engineering software; operational research software; process control systems; expert systems; AI software; educational apps; organizational apps; spreadsheets.
Organized Event: Product of activities aimed at disseminating technical-scientific knowledge by the Graduate Program to academic or general audiences through formally structured events.	Congresses, seminars, festivals, olympiads, competitions, fairs, conventions.
Regulatory Standard or Framework: Guidelines regulating the functioning of public or private sectors, establishing rules for systems, agencies, services, institutions, and companies.	Occupational safety and environmental risk prevention standards; product specifications or process standardization; organizational regulations; practice guides or codes.
Conclusive Technical Report: Concise text containing information about a project or activity from planning to conclusions, highlighting relevance and impact.	Research project reports; technical consulting or advisory reports; contract audit reports.
Manual/Protocol: Set of information, decisions, norms, and rules applied to a specific activity, summarizing essential knowledge or procedures.	Digital communication protocols (HTTPS); Standard Operating Procedures (SOPs).
Translation: A translated work (product) from one language to another, whether literal or free translation.	Translated articles, books, videos, audio materials, or sign language content.
Collection/Archive: Content belonging to a private or public collection, which may be scientific, biological, bibliographic, artistic, photographic, historical, or documentary.	Public and private collections; biological collections.
Technical-Scientific Database: A set of related files containing records about people, places, or things, organized to create meaningful information.	Biological product databases; disease notification systems.
Cultivar: Technological production involving plant varieties that are clearly distinguishable, homogeneous, and stable across generations.	Development, release, and registration of cultivars in RNC/MAPA.
Communication Product: Requires a technological intermediary for communication to occur; a mediated product.	Media programs; communication vehicle programs; social media programs.
Map, Chart, or Similar: Products originating from cartographic studies representing objects, elements, phenomena, or environments.	Charts; maps; photograms.
Confidential Products/Processes: Tangible goods or processes developed through specific procedures, intended for restricted use and validated through confidentiality declarations.	Manufacturing or assembly processes; business management processes; laboratory manipulation techniques; confidential data collection and processing methods.
Taxonomies, Ontologies, and Thesauri: Technical productions that classify, model, and represent concepts and their relationships within a knowledge domain.	Dewey Decimal Classification; Animal Kingdom taxonomy; computing thesaurus; political science ontology.
Innovative Company or Social Organization: A new company or social organization created based on a product, service, or technological process developed by faculty and/or students.	Technomar, a company founded by former master’s and doctoral students from USP’s Numerical Test Tank Laboratory.
Non-Patentable Process/Technology and Product/Material: Technological products or processes that cannot receive formal protection in Brazil due to legal restrictions.	Therapeutic or surgical techniques; new laboratory techniques; biological strains.

After this definition, the Technical and Technological Products (PTT) came to be understood as objects with a high degree of novelty, resulting from research conducted within academic institutions and used to solve problems in companies and in the community at large. In addition, criteria such as impact, innovation, applicability, and complexity were established to distinguish a technological product from a technical product^(10)^.

These differentiation criteria brought important insights for the PMN, since a *technical production* corresponds to the development of personal skills and competencies in professional practice, whereas a *technological product* refers to the creation of an applicable object, derived from scientific knowledge, capable of transforming the reality of a given group. It is believed that clearer definitions have helped better delineate production, guide new and more in depth evaluations, and certainly provide new directions for strengthening the PMN^(10)^.

Another point that deserves reflection concerns the current landscape of technological innovation research in Nursing in Brazil, in a context marked by the lack of significant investment in the health sector in recent decades, despite the implementation of the National Policy on Science, Technology, and Innovation in Health since 2008^(12)^.

Therefore, it is essential to develop strategies and public policies that stimulate technological innovation in Nursing, ensuring freedom and autonomy for research. It is necessary to create approaches that overcome the barriers that hinder the engagement of health professionals - whether in hospitals, clinics, home care, or community settings - with innovation practices and technological productivity, enabling these innovations to be applied and integrated into daily clinical practice and continuous care^(12)^.

## FINAL CONSIDERATIONS

The reflections presented show that the Professional Master’s in Nursing (MPE) has contributed significantly to the practical training of nurses at all levels of care and, in addition to supporting the quality of health services, strengthens Nursing both as a profession and as a scientific field. It is evident that the trajectory of the MPE is recent and challenging, and that its growth and consolidation depend on the quality of the products generated in dissertations, with the purpose of expanding, formulating, and developing new technologies in the health field.

## Data Availability

The research data are available within the article.
