# Supplementary material for: History of Graduate Education in Nursing and the Development of Technological Products in the Professional Master’s Program
Source: Rev Bras Enferm. 2026 Aug 21;79:e20250203. doi: 10.1590/0034-7167-2025-0203 (PMC13506211; doi:10.1590/0034-7167-2025-0203)
Supplement: Translation

# Translation: Histórico da Educação de Pós-Graduação em Enfermagem e Desenvolvimento de Produtos Tecnológicos no Mestrado Profissional

**DOI:** 10.1590/0034-7167-2025-0203pt

**Authors:** Pedro Leite de Melo, Alessandra Matheus Domingos, Daiana Kloh Khalaf, Marina de Góes Salvetti, Maria do Perpétuo Socorro de Sousa Nóbrega

**Affiliations:** I Universidade de São Paulo. São Paulo, São Paulo, Brasil; II Universidade Federal do Paraná. Curitiba, Paraná, Brasil

**Keywords:** Educação de Pós-Graduação em Enfermagem, Desenvolvimento de Produtos Tecnológicos, Pesquisa Científica, Enfermagem, Inovação.

## Abstract

**Objetivos:**

refletir sobre a trajetória e a produção técnica e tecnológica do Mestrado Profissional em Enfermagem.

**Métodos:**

estudo teórico-reflexivo, desenvolvido a partir de levantamento bibliográfico e documental identificado no período de setembro a novembro de 2024, por meio de buscas nas bases *Medical Literature Analysis and Retrievel System Online*, Literatura Latino-americana e do Caribe em Ciências da Saúde, *Scientific Electronic Library Online, Web of Science, Excerpta Medica Database*, e documental, a partir de levantamento de documentos oficiais do Ministério da Educação relacionados às avaliações dos Programas de Pós-graduação, de informações do website da CAPES e da Plataforma Sucupira.

**Resultados:**

após a leitura reflexiva e organização do material selecionado, originou-se a categoria Trajetória histórica, produção técnica e tecnológica do Mestrado Profissional em Enfermagem.

**Considerações Finais:**

os Mestrados Profissionais em Enfermagem ainda enfrentam desafios para seu fortalecimento quanto à sua identidade/objetivo, processo metodológico de construção de produtos, implantação e impacto aos serviços de saúde.

## INTRODUÇÃO

O Mestrado Profissional em Enfermagem (MPE) foi introduzido no Brasil no final da década de 1990, com o objetivo de formar enfermeiros altamente qualificados, aptos para conduzir uma prática avançada e transformadora com vistas a atender demandas sociais, organizacionais e do mercado de trabalho contemporâneo^(1)^.

A Pós-Graduação Stricto Sensu desde a sua idealização tem exercido uma influência vista como primordial em todos os campos de atuação do conhecimento, inclusive na enfermagem, contribuindo fortemente para a evolução da profissão como ciência, e consequentemente no aperfeiçoamento da prática assistencial dos enfermeiros. Este avanço e transformação na prática destes profissionais estão intrinsecamente relacionados ao processo educativo e à qualificação profissional. Nesse sentido, a formação em nível de mestrado e doutorado oferecida não apenas expande a base teórica dos enfermeiros, mas também desenvolve competências práticas e estratégicas essenciais para lidar com os desafios atuais da enfermagem^(2)^.

O Plano Nacional de Pós-Graduação entre 2011 e 2020 registrou uma expansão expressiva dos cursos de Mestrado Profissional em Enfermagem, atingindo 24 programas em 2019, e aprovando dois cursos de doutorado profissional. No entanto, apesar desse progresso, o investimento em inovação tecnológica ainda permanece limitado, impactado por cortes orçamentários do setor público que afetam o financiamento dos programas de pós-graduação^(3)^.

Outro ponto a ser discutido deve-se ao fato de os enfermeiros não terem conhecimentos técnicos e científicos adequados para realizar pesquisas sobre produtividade tecnológica, devido à formação deficiente nessa área e à dificuldade no desenvolvimento de pesquisas que resultem em produção tecnológica, por se tratar de um modelo novo de produção intelectual na enfermagem^(4)^.

No cotidiano tanto pessoal quanto profissional, os enfermeiros estão constantemente em contato com novas tecnologias, e quanto mais rapidamente assimilam e dominam essas inovações para aplicá-las em benefício do ser humano, maiores serão os avanços em sua prática profissional. Nesse contexto, é essencial que os enfermeiros tenham acesso e utilizem pesquisas científicas e tecnológicas para contribuírem para a melhoria da qualidade do cuidado prestado^(5)^.

O MPE não se limita à capacitação do mestrando, mas exige a realização de uma investigação científica relacionada à sua área de atuação. No entanto, não basta apenas analisar os desafios da prática profissional - é fundamental que esses estudos resultem em propostas de solução, concretizadas em diferentes formas de produção tecnológica^(6)^.

A produção tecnológica ou técnica faz referência ao trabalho desenvolvido por docentes permanentes e discentes que não se enquadram como produção científica. Seu valor é reconhecido pela interação entre a academia e a comunidade, tendo como resultado as aplicações práticas e inovadoras para demandas/necessidades reais^(7)^.

## OBJETIVOS

Refletir sobre a trajetória e a produção técnica e tecnológica do MPE.

## MÉTODOS

Estudo teórico-reflexivo sobre o percurso histórico dos MPE, desde a Portaria nº 80/16/12/1998, emitida pela Coordenação de Aperfeiçoamento de Pessoal de Nível Superior (CAPES), que estabelece as diretrizes para a criação e funcionamento^(8)^.

O material bibliográfico foi identificado no período de setembro a novembro de 2024 por meio de buscas nas bases MEDLINE/PUBMED (*Medical Literature Analysis and Retrievel System Online*), LILACS (Literatura Latino-americana e do Caribe em Ciências da Saúde), SCIELO (*Scientific Electronic Library Online*), WoS (*Web of Science*) e EMBASE (*Excerpta Medica Database*), com os descritores: Educação de Pós-Graduação em Enfermagem, Desenvolvimento de Produtos Tecnológicos, Pesquisa científica, Enfermagem, e Inovação. O material documental foi identificado a partir de documentos oficiais do Ministério da Educação (ME) relacionados às avaliações dos Programas de Pós-Graduação, de informações do website da CAPES (https://www.gov.br/capes/pt-br) e da Plataforma Sucupira (https://sucupira.capes.gov.br/), no mesmo período.

As informações extraídas dos estudos foram organizadas pelos autores dessa reflexão durante todo o processo de seleção, leitura e análise dos textos, seguido de elaboração de notas teórico-reflexivas sobre os principais pontos para tomada de decisão e interpretação analítica.

As experiências dos autores sobre a temática, sustentadas no aporte teórico sobre os desafios encontrados para consolidação e caracterização dos produtos técnicos/tecnológicos do MPE, e a leitura atenta, em busca de informações sobre sua trajetória, permitiram a elaboração do estudo.

As reflexões apresentadas foram desenvolvidas com base nos aspectos teóricos e conceituais abordados pelos estudos, assim como nas impressões dos autores, derivadas de sua compreensão do tema e experiência empírica. Por não envolver coleta de dados com seres humanos, não houve necessidade de análise ética. Todavia, foram considerados procedimentos para garantir que os estudos primários aplicados fossem respeitados eticamente.

## RESULTADOS

### Trajetória histórica, produção técnica e tecnológica do Mestrado Profissional em Enfermagem

A CAPES classifica os MPE como uma modalidade de Pós-Graduação *Stricto Sensu* voltada à capacitação profissional em diversas áreas do conhecimento, por meio do estudo de técnicas, processos e temas alinhados às demandas do mercado de trabalho^(9)^. A partir da Portaria nº 80 de 16/12/1998, foi criado no país, em 2001, o primeiro MPE com enfoque em obstetrícia, vinculado à Universidade Federal de São Paulo, que após formar quatro mestres encerrou suas atividades em 2004.

O Quadro 1 ilustra a linha do tempo do percurso dos MPE de 2003 até 2019, com o mapa de 24 cursos de acordo com o Relatório CAPES 2017-2020^(10)^.

**Quadro 1:** Linha do tempo dos Mestrados Profissionais em Enfermagem de 2003 a 2019, São Paulo, São Paulo, Brasil

Programa de Pós-graduação Ano de criação	Instituição de Ensino Superior	Área de concentração dos programas
Programa de Pós-Graduação Mestrado Profissional em Enfermagem Assistencial - 2003	Universidade Federal Fluminense	Processos de cuidar em enfermagem
Programa Pós-graduação em Enfermagem - 2006	Universidade Estadual Paulista Júlio de Mesquita Filho em Botucatu	Enfermagem
Gestão do cuidado em Enfermagem - 2010	Universidade Federal de Santa Catarina	Gestão do cuidado em saúde e enfermagem
Programa Pós-graduação em Enfermagem - 2011	Universidade Federal do Paraná	Prática Profissional de Enfermagem
Programa Pós-graduação em Enfermagem - 2011	Universidade Federal do Espírito Santo	Cuidado e Administração em Saúde
Programa Pós-graduação em Enfermagem - 2011	Universidade do Vale do Rio dos Sinos	Práticas do cuidado em enfermagem
Programa Pós-graduação em Saúde da Família- 2011	Centro Universitário UNINOVAFAPI	Saúde da família
Programa Pós-graduação em Enfermagem -2012	Universidade Estadual de Feira de Santana	Enfermagem
Programa Pós-graduação Ciências para Saúde - 2012	Fund. Ensino e Pesquisa em Ciências de Saúde	Qualidade da Assistência à Saúde
Programa Pós-graduação Saúde e Tecnologia no Espaço Hospitalar - 2013	Universidade Federal do Estado do Rio de Janeiro	Saúde e tecnologia no espaço hospitalar
Programa Pós-graduação Tecnologia e Inovação em Enfermagem - 2013	Universidade Federal de São Paulo/Ribeirão Preto	Tecnologias do Cuidar em Enfermagem
Programa Pós-graduação Enfermagem na Atenção Primária em Saúde no SUS - 2013	Universidade de São Paulo	Cuidado em atenção primária em saúde
Programa Pós-graduação em Enfermagem -2014	Sociedade Beneficente Israelita Brasileira Albert Einstein	Ensino e Saúde
Programa Pós-graduação em Saúde da Família -2015	Faculdade Nova Esperança	Gestão e Tecnologias do Cuidado em Saúde
Programa Pós-graduação em Enfermagem -2015	Universidade Federal de Ciências da Saúde de Porto Alegre	Enfermagem
Programa Pós-graduação Saúde materno infantil - 2015	Universidade Franciscana	Saúde Materno Infantil
Programa de Pós-graduação Tecnologia e Inovação em Enfermagem - 2016	Universidade de Fortaleza	Tecnologias do Cuidar em Enfermagem
Programa Pós-graduação em Gerontologia -2016	Universidade Federal da Paraíba	Gerontologia
Programa Pós-graduação Informática em Saúde - 2016	Universidade Federal de Santa Catarina	Informática em saúde
Programa Pós-graduação em Enfermagem na Atenção Primária à Saúde - 2017	Universidade do Estado de Santa Catarina	Promoção da saúde na Atenção Primária
Programa Pós-graduação em Saúde e Sociedade - 2017	Universidade Federal Rio Grande do Norte	Saberes e Práticas em Saúde e Educação
Programa de Pós-graduação em Enfermagem em Saúde Pública - 2019	Universidade do Estado do Amazonas	Práticas de Enfermagem em Saúde Pública na Amazônia
Programa de Pós-graduação em Enfermagem no Contexto Amazônico - 2019	Universidade Federal do Amazonas	Prática clínica avançada na enfermagem amazônica
Programa de Pós-graduação em Enfermagem - 2019	Universidade do Estado de Santa Catarina	Enfermagem

Desde 2014, o Conselho Federal de Enfermagem (COFEN) inclui nos planos trienais propostas de financiamento para MPE, voltados aos enfermeiros que atuam no Sistema Único de Saúde, para aprimorar a qualidade da assistência em saúde^(9)^. Em meados de 2016 lançou o primeiro edital para o MPE, e destinou recursos para Instituições de Ensino Públicas e privadas interessadas em promover pesquisas na área de Sistematização da Assistência de Enfermagem.

No ano seguinte, CAPES e COFEN estabeleceram uma comissão avaliadora conjunta que selecionou 16 programas e distribuiu 140 vagas. Além disso, nos 20º, 21º e 22º Congressos Brasileiros dos Conselhos de Enfermagem, realizados entre 2017 e 2019, foram incluídas mesas redondas sobre o MPE na programação científica, para discutir aspectos do funcionamento^(9)^.

A produção técnica/tecnológica dos MPE foi avaliada pela primeira vez na avaliação quadrienal dos programas (2013-2016) quanto a sua tipologia, e mostrou que ainda há desafios para sua classificação com vistas a garantir que de fato contribuam efetivamente para a qualidade dos serviços de saúde^(10)^.

O MPE está em processo de consolidação, especialmente quanto à elaboração e aprimoramento da produção técnica e tecnológica, pois ainda apresentam dificuldade na classificação de seus produtos, sendo necessário, na maioria das vezes, avaliar o material na íntegra para entender a tipologia e categorizá-la corretamente. Outro ponto em relação à qualificação do MPE diz respeito a sua identidade, objetivo, processo metodológico de construção de produtos e de sua implantação, bem como ao impacto que trazem aos serviços de saúde^(11)^.

Antes de 2012, não havia um *Qualis* tecnológico para avaliar a produção do MPE. A avaliação era feita com base nos critérios e métricas do Mestrado Acadêmico, focado na produção intelectual^(6)^. Para dirimir essa problemática, a CAPES elaborou em 2012 um roteiro de orientação para a Comissão Assessora melhorar as informações na Plataforma Sucupira, abordando seis quesitos: proposta, corpo docente, corpo discente, teses e dissertações/trabalhos de conclusão, produção intelectual e inserção social^(7)^.

Com o crescimento do MPE ao longo dos últimos anos, um número expressivo de produtos técnicos e tecnológicos foram desenvolvidos pelos enfermeiros, com a proposição de oferecer inovações para a prática, garantir segurança e qualidade no cuidado, e valorizar a expertise profissional^(5)^.

Para lidar com esse complicador, e qualificar o MPE, a Diretoria de Avaliação da CAPES instituiu em 2016 o Grupo de Trabalho *Qualis* Técnico e Tecnológico (GT-PTT), que reuniu 64 produções técnicas disponíveis na Plataforma Sucupira (quadrienal 2013-2016), e analisou e definiu 21 tipos de Produção Técnica Tecnológica, aprovadas em 2019 pelo Conselho Técnico-Científico da Educação Superior^(10)^, conforme Quadro 2.

**Quadro 2:** Caracterização das produções técnicas/tecnológicas, São Paulo, São Paulo, Brasil

Tipo de produção técnica e Definição	Exemplos
Produto bibliográfico: Tem como finalidade a divulgação do conhecimento produzido dentro das academias.	Artigo publicado em revista técnica; Resenha ou crítica; Artigo em jornal ou revista de divulgação, entre outros.
Ativos de propriedade intelectual: São recursos que agregam valor às marcas e aos produtos desenvolvidos, além de permitir os direitos autorais.	Patente de invenção, patente de modelo de utilidade, certificado de adição; Marca, Desenho Industrial, Indicação Geográfica; Topografia de Circuitos Integrados.
Tecnologia Social: Métodos, processos ou produtos desenvolvidos e aplicados com a população, com o intuito de melhorar as condições de vida na comunidade. Precisa ser simples, de baixo custo e de fácil aplicabilidade.	Projeto de Leitura nos Terminais de ônibus, Técnicas alternativas de agricultura, educação em saúde bucal em determinados grupos populacionais.
Curso para formação profissional: Aglomerado de conteúdos estabelecidos de acordo com as competências requeridas pela formação profissional, em conformidade com os objetivos do Programa de Pós-Graduação.	Formação contínua para profissionais com vínculo institucional; Oferta especial para profissionais vinculados a projetos de pesquisa.
Produto de Editoração: Produto de editoração resulta de atividade editorial de processos de edição e publicação de obras de ficção e não-ficção. Compreende planejar e executar, intelectual e graficamente, livros, enciclopédias, preparar textos, ilustrações e diagramação.	Mídia impressa (jornal, revista, livro, panfleto, cartaz, etc.), eletrônica (e-books, mídias interativas) ou digital (internet, celular).
Material didático: Produto de apoio ou suporte com fins didáticos na mediação de processos de ensino e aprendizagem em diferentes contextos educacionais.	Impresso: coleções; livro didático e paradidático; guias; mapas temáticos; jogos educativos, entre outros.
Software/Aplicativo (Programa de computador): *Software* é um conjunto de instruções ou declarações a serem usadas direta ou indiretamente por um computador a fim de obter um determinado resultado.	Programas de Simulação, softwares de engenharia, softwares de pesquisa operacional, controle de processos, sistemas especialistas, softwares de inteligência artificial, aplicativos educacionais, aplicativos utilizados em ambiente organizacional, planilhas eletrônicas, etc.
Evento Organizado: Produto da atividade de divulgação e/ou propagação do conhecimento técnico-científico pelo Programa de Pós-Graduação para público acadêmico ou geral por meio de atividades formalmente concebidas.	Congresso, seminário, festival, olimpíada, competição, feira ou convenção realizada pelo Programa de Pós-Graduação.
Norma ou Marco regulatórias: São diretrizes que regulam o funcionamento do setor público ou privado. Tem por finalidade estabelecer regras para sistemas, órgãos, serviços, instituições e empresas, com mecanismos de regulação, compensação e penalidade.	Norma regulamentadora em segurança e saúde no trabalho e prevenção de riscos ambientais; especificação de produto ou padronização de processo; regulamento ou norma organizacional relacionada à negócios, à órgão governamental, à associação comercial ou profissional, à grupo de consumidores; guia ou código de prática.
Relatório técnico conclusivo: Texto elaborado de maneira concisa, contendo informações sobre o projeto ou atividade realizada, desde seu planejamento até as conclusões. Indica em seu conteúdo a relevância dos resultados e conclusão em termos de impacto social ou econômico e a aplicação do conhecimento produzido.	Relatório de projeto de pesquisa; Relatório de assessoria e consultoria técnica e de auditoria de contratos.
Manual/Protocolo: Conjunto das informações, decisões, normas e regras que se aplica a determinada atividade, que encerra os conhecimentos básicos de uma ciência, uma técnica, um ofício, ou procedimento. Pode ser um guia de instruções que serve para o uso de um dispositivo, para correção de problemas ou para o estabelecimento de procedimentos de trabalho. No formato de compêndio, livro, guia pequeno ou um documento, normativa, impresso ou digital, que estabelece como se deve atuar em certos procedimentos.	Protocolos de comunicação digital (https), Procedimento Operacional Padrão (POP - documento organizacional que traduz o planejamento do trabalho a ser executado, sendo uma descrição detalhada de todas as medidas necessárias para a realização de uma tarefa).
Tradução: Obra traduzida (produto) de uma língua para outra, independentemente se foi resultado de uma tradução literal ou de tradução livre.	Obras traduzidas como artigos, livros e similares, vídeos, áudios ou sinais.
Acervo: Conteúdo de uma coleção privada ou pública, podendo ser de caráter científico, biológico, bibliográfico, artístico, fotográfico, histórico, documental, misto ou qualquer outro.	Coleções públicas e privadas; Coleções biológicas.
Base de dados técnico-científica: Conjunto de arquivos relacionados entre si com registros sobre pessoas, lugares ou coisas. São coleções organizadas de dados que se relacionam de forma a criar algum sentido (Informação) e dar mais eficiência durante uma pesquisa ou estudo.	Banco de dados de produtos biológicos, sistema de agravos de notificação.
Cultivar: Produção tecnológica na qual a variedade de qualquer gênero ou espécie vegetal superior que seja claramente distinguível de outras cultivares conhecidas por margem mínima de descritores, por sua denominação própria, que seja homogênea e estável quanto aos descritores através de gerações sucessivas e seja de espécie passível de uso pelo complexo agroflorestal.	Desenvolvimento, Lançamento e Registro de cultivares no RNC/MAPA, que poderão ser geradas através de cruzamento ou originadas por mutação, transformação ou edição gênica.
Produto de comunicação: Implica na existência de um intermediário tecnológico para que a comunicação se realize. Trata-se, portanto, de produto midiatizado. Mídia compreende o conjunto das emissoras de rádio e de televisão, de jornais e de revistas, do cinema e das outras formas de comunicação de massa, bem como, das recentes mídias sociais em suas diversas plataformas.	Programas de mídia; Programas de veículos de comunicação; Programas de mídia social.
Carta, mapa ou similar: Produtos com origem em estudos cartográficos, representando objetos, elementos, fenômenos e ambientes	Carta; Mapa; Fotograma.
Produtos/Processos em Sigilo: Produto: Bens físicos/tangíveis obtidos por combinação de ideias, que possam ser materializados ou produzidos por um determinado processo de fabricação, destinados ao uso restrito e comprovado por meio de declaração de sigilo. Processo: Conjunto ordenado de procedimentos/operações/atividades que objetivam transformar uma Entrada (insumos materiais ou não) em uma Saída desejável (produtos, serviços), com repetibilidade, previsibilidade e confiabilidade, destinados ao uso restrito e comprovado por meio de declaração de sigilo.	Processos de fabricação ou montagem, processos de gestão empresarial, técnicas de manipulação laboratorial, técnicas de coleta e tratamento de dados, considerados sigilosos.
Taxonomias, Ontologias e Tesauros: São produções técnicas que “classificam, modelam e representam conceitos e seus relacionamentos pertinentes a um domínio do conhecimento”.	Classificação Decimal Dewey (CDD), utilizada em bibliotecas; Taxonomia do Reino Animal; Taxonomia de Lineu (biologia); Taxonomia dos objetivos educacionais (educação); Tesauro da Ciência da Computação (computação); Tesauro do movimento corporal infantil (anatomia); Ontologia da ciência política (sociologia); Ontologia da termodinâmica (física).
Empresa ou Organização Social Inovadora: Uma nova empresa ou organização social formada com base em produto, serviço ou processo tecnológico desenvolvido por docentes e/ou discentes no âmbito do Programa de Pós-Graduação.	Technomar, empresa formada por dois ex-alunos de mestrado e doutorado do Laboratório Tanque de Prova Numérico (TPN) da Escola Politécnica da USP.
Processo / Tecnologia e Produto / Material não patenteáveis: Produtos e/ou processos tecnológicos que, por impedimentos legais, não apresentam um mecanismo formal de proteção em território brasileiro, incluindo quaisquer ativos de propriedade intelectual, como, por exemplo, métodos terapêuticos e cirúrgicos.	Nova técnica de inserção de ponte de safena, novas formas de exercício físico condicionantes para atleta, cepas da área biológica.

Após essa definição, os PTT passaram a ser objetos com um grau elevado de novidade, fruto das pesquisas realizadas nas academias, e usados para resolução de problemas nas empresas e na comunidade em geral. Ademais, estabeleceram-se critérios como impacto, inovação, aplicabilidade e complexidade para distinguir um produto tecnológico de um produto técnico^(10)^.

Estes critérios de diferenciação trouxeram respostas importantes para os MPE, uma vez que uma produção técnica corresponde ao desenvolvimento de habilidades e competências pessoais na prática profissional, e o produto tecnológico diz respeito à criação de um objeto aplicável, fruto do conhecimento científico, que permitirá transformar a realidade de um determinado grupo. Acredita-se que definições mais claras têm ajudado a definir melhor a produção e conduzir novas e mais aprofundadas avaliações, e certamente trazer novos direcionamentos para qualificação do MPE^(10)^.

Outro ponto que merece reflexão diz respeito ao atual panorama das pesquisas em inovação tecnológica na Enfermagem no Brasil, em um cenário caracterizado pela falta de investimentos significativos na área da saúde nas últimas décadas, apesar da implementação da Política Nacional de Ciência, Tecnologia e Inovação em Saúde desde 2008^(12)^.

Portanto, é fundamental desenvolver estratégias e políticas públicas que estimulem a inovação tecnológica na Enfermagem, garantindo liberdade e autonomia para a pesquisa. É preciso criar abordagens que superem as barreiras que dificultam a aproximação dos profissionais de saúde, em ambientes como hospitais, clínicas, domiciliares ou comunitários, com a prática de inovação e produtividade tecnológica, permitindo que essas inovações sejam aplicadas e integradas à prática clínica diária e ao cuidado contínuo^(12)^.

## CONSIDERAÇÕES FINAIS

As reflexões apresentadas mostram que o MPE tem contribuído significativamente para a formação prática dos enfermeiros em todos os níveis da assistência e, além de colaborar para a qualidade dos serviços de saúde, fortalece a Enfermagem no campo profissional e como ciência. Nota-se que a trajetória é recente e desafiadora para o MPE, e que seu crescimento e fortalecimento implica em bons produtos gerados nas dissertações, no propósito de ampliar, formular e desenvolver novas tecnologias na área da saúde.

## Data Availability

Os dados de pesquisa estão disponíveis no corpo do artigo.
